# Reducing anxiety and depression in infertility among Nigerian women: an exploratory psycho-educational intervention trial (RADIANT) study protocol

**DOI:** 10.11604/pamj.2021.39.43.23703

**Published:** 2021-05-18

**Authors:** Folasade Adenike Bello, Josephine Oluyemisi Adeolu, Jibril Omuya Abdulmalik, Rukiyat Adeola Abdus-salam, Olayinka Abimbola Egbokhare, Oluwaseun Aramide Otekunrin, Olatunde Olayinka Ayinde, Ade Fatai Adeniyi

**Affiliations:** 1 Department of Obstetrics and Gynecology, University of Ibadan, Ibadan, Nigeria,; 2 Department of Community Medicine, University College Hospital, Ibadan, Nigeria,; 3 Department of Psychiatry, University of Ibadan, Ibadan, Nigeria,; 4 Department of Obstetrics and Gynecology, Adeoyo Maternity Teaching Hospital, Ibadan, Nigeria,; 5 Department of Communication and Language Arts, University of Ibadan, Ibadan, Nigeria,; 6 Department of Statistics, University of Ibadan, Ibadan, Nigeria,; 7 Department of Physiotherapy, University of Ibadan, Ibadan, Nigeria

**Keywords:** Infertility, anxiety, depression, Nigeria

## Abstract

Rationale: high premium is placed on infertility in Nigerian culture. Data is limited on its association with emotional problems in Nigeria. Aims: to develop content for a culturally relevant and cost-effective psychoeducational intervention package and to evaluate its effectiveness for reducing symptoms of anxiety and depression. Sample size estimate: Methods and design: a multi-method study design including development and validation (which includes focus group discussions) of an audio-visual tool which will serve as the intervention in a randomized controlled trial. Data will be analyzed with interim and survival analyses. Population studied: one hundred and 138 (68 per group) infertile women attending infertility clinic in Ibadan. Study outcomes: anxiety and depressions scores assessed with the hospital depression and anxiety scale (HADS) at 0, 3 and 6 weeks. Discussion: it is hoped that the use of the audio-visual tool will improve participants depression and anxiety scores and that the tool will be used for education in routine clinic use and community awareness on psychosocial effects of infertility.

## Study protocol

### Introduction

Infertility is defined as “a disease of the reproductive system defined by the failure to achieve a clinical pregnancy after 12 months or more of regular unprotected sexual intercourse” [1]. From Demographic and Health Surveys (DHS) from developing countries, one in four couples is affected by infertility [2]. High premium is placed on childbirth in Nigeria; all couples are traditionally expected to procreate. Nigeria's DHS does not report infertility prevalence, however, rural community surveys reported prevalence of 8.7% [3] and 30.3% [4]. Infertility constitutes 24-59% of gynecological consultations in Nigeria [5-8].

Infertile women have feelings of loss, grief, anger, sadness, shame, self-blame and lack of femininity [9,10], which predict actual psychopathology [11]. Though a couple's problem, the woman bears the larger burden of the associated stigma, due to socio-cultural beliefs that reproduction is the woman´s primary responsibility, and a failure is her failing [12,13]. Studies show that infertile women are more likely to be economically disadvantaged [14], suffer psychological distress [15], be socially stigmatized [12,14,16] and suffer intimate partner violence [17], than fertile women. The resulting marital disharmony may lead to divorce, for which the woman is blamed [18]. Infertile men may also have more anxiety and depression symptoms than fertile men [19], but these appear to be less than those suffered by their partners [20].

Nigerian studies have suggested counselling and psychological interventions to ameliorate the documented psychosocial aspects of infertility [15,20,21]. However, no interventional studies were found on the subject. Evidence from outside of sub-Saharan Africa suggests that psychosocial interventions for infertile couples are effective [22-24]. An innovative approach to intervention delivery is the use of eHealth programming, “the use of emerging interactive technologies [e.g. internet, CD-ROMs etc.] to enable health improvement and health care services” [25]. These have the advantage of not requiring facilitators and being reproducible and consistent across time and location.

This study proposes to identify psychosocial and emotional needs in infertile women, develop an interventional ehealth tool and determine its effectiveness in improving the psychological and emotional health of infertile women. Traditional one-on-one counselling as part of on-going infertility evaluation and care is often difficult to perform in the study environment, due to lack of skills in gynecologists and stigma attached to seeing a psychiatrist. Integrating mental health care into regular infertility clinics may also be difficult due to dearth of providers.

The authors hope to employ eHealth to combine theoretic knowledge and coping skills with drama, which is a popular form of local entertainment. This is a concept coined as 'edutainment', “entertainment (as by games, films, or shows) that is designed to be educational” [26]. If effective, this intervention does not require face-to-face counselling. Family and close friends can also watch and can be educated on supporting the infertile couple. The aims of the study are to: 1) develop content for a culturally-relevant and cost-effective psychoeducational intervention package aimed at reducing social and emotional problems among women with infertility in Ibadan, Nigeria; 2) translate the developed content into an audio-visual drama production as a user-friendly tool for the target audience; and 3) evaluate the effectiveness of this psychoeducational intervention for reducing symptoms of anxiety and depression among women with infertility in Ibadan, Nigeria.

### Methods

A multi-method study design will be utilized and implemented in two phases; phase i: development and validation of audio-visual psychoeducational intervention material; phase ii: exploratory randomized controlled trial to test the effectiveness of the intervention. The development of psychoeducational material will be a multi-disciplinary process involving the following specialists - psychiatrist, gynecologist, public health physician and communication and language arts expert. Theatre arts' professionals and students will also be recruited subsequently. The two phases are described below.

**Phase I: development of psychoeducational intervention material:** this will be conducted in the following steps: 1) Content development: a) literature review: a comprehensive review of the literature will be performed and a composite list of emergent themes that affect women with infertility globally and especially in the Nigerian socio-cultural setting, will be developed. This will be independently performed by two units of two members each, with subsequent harmonization of both lists by the research group through discussions and consensus; b) gender consideration: two focus group discussions (FGDs) and several key informant interviews (KIIs) of infertile males (or spouses of infertile women) will be organized to understand the perceptions and experiences of males with respect to issues of infertility. This is important, as only few studies conducted among men were found in literature [20,27], resulting in little being known about male experience. The investigators hope to gain a holistic understanding from both male and female perspectives, which should strengthen the content development process of the psycho-educational intervention; c) specialist input from gynecologists and psychiatrists: the gynecologists will provide a summary sheet about the causes, course, treatment options and outcomes of infertility globally and in the study environment. The psychiatrists will also provide summary points about coping skills and building resilience in the face of life challenges as an essential life skill; d) emergence of the psychoeducational content: the combination of information derived from the three sources above will then be distilled by two units working independently to develop the psychoeducational content. Both teams will then meet to harmonize and resolve areas of disagreement and to produce a final summative material that is arranged into themes. 2) Translation of the developed content into a storyline and script. The content as developed in step 1 will be transformed into a storyline with specific emphasis on effective communication and message impact for the target audience. This will be overseen by the communications and language arts expert. 3) Production of psychoeducational intervention video: the script will be acted by professional actors, as well as students of the Department of Theatre Arts of the University of Ibadan, to produce a short film which will illustrate and deliver the key messages derived from the content. 4) Pre-test and establishment of face validity: the final video produced to deliver the psychoeducational intervention will be pretested and screened to a selection of nurses and doctors (independent of the study team) and 6-10 women with infertility (drawn from a different facility, other than the proposed study sites). Their feedback and views will be sought thereafter for acceptability, critical feedback and comments. The production will be edited as appropriate, following this screening.

### Phase II: randomized controlled trial to test the effectiveness of the psychoeducational intervention

**Study design:** a randomized controlled trial with an allocation ratio of 1: 1 will be utilized.

**Study setting:** 1) University College Hospital (UCH), Ibadan, Nigeria: it serves as a referral tertiary center for Nigeria and sub-Saharan Africa. The study will be at the outpatient gynecology clinic of the hospital; 2) Adeoyo Maternity Hospital (AMH), Yemetu, Ibadan, Nigeria: this is a province-owned secondary health care facility.

**Study subjects:** women attending gynecology clinics in UCH or AMH on account of infertility. Consenting participants will be randomized into the intervention and control arms of the study, respectively.

**Eligibility criteria:** women aged 18 years and above, who have been trying to conceive for at least one year, and who do not have any children.

**Interventions:** consecutive, eligible infertility clinic attendees will be invited to participate in the study and informed consent obtained. Sociodemographic data would be collected via a structured questionnaire. Baseline assessment on participants' psychological and emotional state will be collected via Hospital Anxiety and Depression Scale (HADS) [28]. Thereafter, the participants will be allocated into study groups. Participants in the intervention arm will attend the intervention session on a specified date at the facility. The psychoeducational video will be viewed, and immediate feedback will be obtained. Participants in the control arm will also receive the usual treatment offered all attendees at the gynecology clinics - comprising health talks by public health nurses and explanations and counselling provided by the managing physicians. The study´s primary outcomes are depressive and anxiety symptoms. HADS is a self-assessment scale which has subscales that measure severity of anxiety and depression. It is validated for use in medical outpatient clinics. Participants are followed up at three and six weeks; HADS will be administered at both times. Participants will be counseled on the need for follow-up. Recruited respondents in both arms will receive phone call reminders ahead of scheduled clinic appointments to improve attendance and minimize loss to follow-up.

**Sample size estimation:** the minimum sample size was determined using the formula for comparing two means i.e.

$$\text{n=}\frac{2{\left[Z_{1-\alpha /2}+Z_{1-\beta }\right]}^{2}}{\Delta^{2}}$$

where Δ, effect size =

$$\Delta =\frac{\mu_{1}-\mu_{1}}{\sigma }$$

where Z1-α/2 [α] was set at 5% i.e. 1.96; Z1-β [β] was set at 20% i.e. power at 80% = 0.84 [29]. A moderate effect size of 0.53 was used. A minimum sample size of 57 was calculated. Attrition rate of 20% was incorporated in the calculation and sample size was estimated at 68 women in each group.

**Randomization procedure:** a randomization list of randomly permuted blocks of treatment assignments in blocks of 4 will be prepared by the study statistician, using a computer-generated table of random numbers. The research assistant will open the sealed envelope containing the assigned group after baseline HADS has been administered. The clinical staff recruiting participants and the data analyst will be blinded, as he will receive two spreadsheets of data without knowing which is the intervention arm.

**Figure 1:**
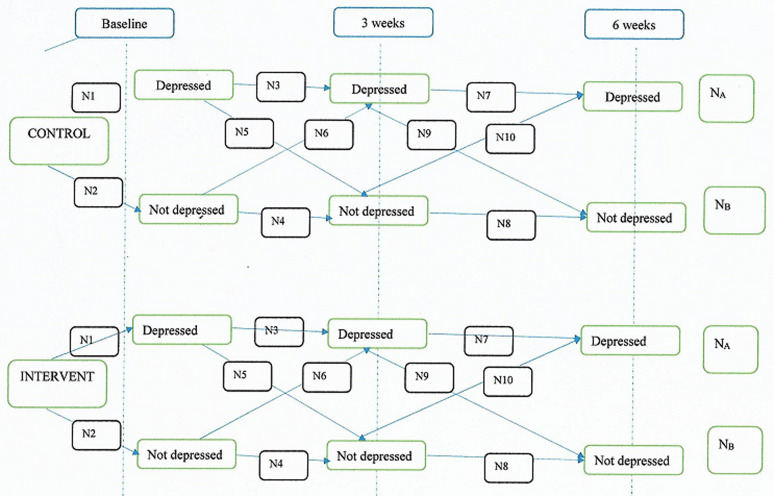
program evaluation and review technic (PERT) diagram showing flow of participants into cases or non-cases

**Data management and analysis:** the data collected will entered into the computer and analysed using IBM SPSS version 23.0. Interim analysis will be carried out using Mann-Whitney-U test to compare independent samples of the intervention and control groups at 0, 3 and 6 weeks. Survival analysis will also be carried out at each time to assess number of cases of depression or anxiety that transited to non-cases (Figure 1). A data monitoring committee is not necessary as there are no interventions bordering on patient safety. There are no issues that might necessitate termination of trial. Malfeasance or adverse events are not applicable in a trial that constitutes audiovisual expert education as the intervention.

**Ethical considerations:** ethical approval was obtained from the University of Ibadan/University College Hospital Ethical Committee (Ref: UI/EC/17/0003). Protocol amendments will be communicated to the ethics committee and written approval obtained before commencing. Clinical care providers in the clinic will recruit the participants and obtain assent, then a trained research assistant will obtain informed consent (Annex 1). Anonymized data will be obtained from participants in privacy and data collected will be kept in a secure locked cabinet in an office. Data entered on to the study computer will be password-protected. Personal information will only be recorded in order to send reminders and invitations for follow up sessions. The investigators will have access to the final data set and will not disclose information to other parties.

**Trial registration:** the trial is registered with Pan African Clinical Trial Registry [PACTR201901892865101].

**Reporting of trial results:**Table 1 will describe the demographics of the participants in the intervention and control groups, while Table 2 will show the proportion of cases to non-cases. The changes in these over 6 weeks will be documented in Table 3 and Figure 1. Trial findings will be communicated in a written report to sponsors, presented at a gynecology conference and published in a per-reviewed journal. The authors will be those who have contributed to the conception, design, data acquisition and writing of the manuscript. The full protocol and dataset will be available by contacting the corresponding author.

**Table 1:** distribution of socio-demographic characteristics of participants

Variables	Classes	Intervention group No [%]	Control group No [%]	p-value
Age	< 35 years			
	≥ 35 years			
Marital status	Married			
	Unmarried			
Education	None			
	Primary/ secondary			
	Tertiary			
Occupation	Unemployed			
	Unskilled			
	Skilled			
Previous pregnancies	Yes			
	No			
Identified cause of infertility	Self			
	Male partner			
	Neither/both/ unknown			
Length of infertility	≤ median			
	> median			
Average monthly family income*	≤ median			
	> median			

**Table 2:** participants diagnosed of depression and anxiety by HADS

Variables	Classes	Anxiety				Depression			
		Control group		Intervention group		Control group		Intervention group	
		Cases No. [%]	Non-cases No. [%]	Cases No. [%]	Non-cases No. [%]	Cases No. [%]	Non-cases No. [%]	Cases No. [%]	Non-cases No. [%]
Age	< 35 years								
	≥ 35 years								
Marital status	Married								
	Unmarried								
Education	None								
	Primary/ secondary								
	Tertiary								
Occupation	Unemployed								
	Unskilled								
	Skilled								
Previous pregnancy	Yes								
	No								
Identified cause of infertility	Self								
	Male partner								
	Both/ unknown								
Length of infertility	< median								
	≥ median								
Average monthly family income	< median								
	≥ median								

**Table 3:** comparison of the HADS scores between intervention and control groups at 0, 3 and 6 weeks

	HADS at baseline				HADS at 3 weeks				HADS at 6 weeks			
	Intervention group	Control group	X^2^ test	p-value	Intervention group	Control group	X^2^ test	p-value	Intervention group	Control group	X^2^ test	p-value
Cases												
Non-cases												
Total												

X2 test: Chi square test

## Annex

**Annex 1**: reducing anxiety and depression in infertility among Nigerian women: an intervention trial [radiant] study (PDF- 61.2 KB)
